# Broad-Spectrum Antivirals and Antiviral Combinations: An Editorial Update

**DOI:** 10.3390/v14102252

**Published:** 2022-10-14

**Authors:** Denis Kainov, Valentyn Oksenych

**Affiliations:** 1 Department for Clinical and Molecular Medicine (IKOM), Norwegian University of Science and Technology, 7491 Trondheim, Norway; 2 Institute of Technology, University of Tartu, 50090 Tartu, Estonia; 3 Institute of Clinical Medicine, University of Oslo, 0318 Oslo, Norway

Our Special Issue received a great deal of attention, and several important papers have recently been added to it. Thus, we are pleased to extend our previous Editorial [1] and to highlight all the papers of the issue.

In this Special Issue, several papers are focused on broad-spectrum antivirals (BSAs). BSAs could be referred to as antivirals that target many viruses of the same family (pan-family inhibitors) or viruses belonging to different viral families (cross-family inhibitors). An important direction in the development of BSAs is to expand their antiviral activity, i.e., to find new targets [1,2]. 

A paper by Wald et al. expanded the activity of ivermectin to Usutu virus, which is a mosquito-borne arbovirus harmful to birds [3]. Another paper highlighted nafamostat, a serine protease inhibitor, as a drug that could suppress coronavirus-mediated ribosomal frameshift [4]. The article is followed by a Comment [5], pointing out the difference in the mechanisms of action of nafamostat in Vero E6, Calu-3, and A549 cell lines. A review published in this Special Issue summarized data on the structure of flavivirus NS2B-NS3 and attempted to develop antivirals based on available cellular and in vivo models [6]. Some of these antivirals could possess pan-family BSA activity.

An immunoglobulin (Ig)-based approach is highlighted in the paper published by Ravlo et al. [7]. This approach could be efficient against emerging and re-emerging viral infections, because of the reduced cost and time of their development, in contrast to the development of monoclonal antibodies. However, there are advantages and disadvantages of Ig. For example, polyclonal IgYs can neutralize different viral variants in vitro at relatively low concentrations; however, higher concentrations or further purification and enrichment of specific IgYs are needed to neutralize the viruses in vivo.

Many viruses have developed multiple mechanisms to escape BSA actions. Therefore, BSAs are combined with other antiviral agents into BSA-containing drug combinations (BCCs). Three studies aiming to identify efficient BCCs have been published in the Special Issue [8,9,10].

In particular, Ianevski et al. reported new BCCs containing pleconaril, vemurafenib, and rupintrivir for the treatment of echovirus 1 infection [10]. It was noted that lower doses of drugs were needed to inhibit the virus replication than for monotherapies, suggesting a lower risk of side effects.

Two other papers reported interferon (IFN)-alpha-containing combinations against SARS-CoV-2 virus. These cocktails were more efficient than monotherapies [8,9]. IFNs are natural BSAs. However, type I IFNs were insufficient to block and eliminate viruses in vitro. It was shown that IFN-alpha can be combined with convalescent serum, cycloheximide, camostat, EIDD-2801, remdesivir, or nafamostat to synergistically abrogate or ablate viral replication in vitro. Interestingly, the combination of nafamostat and IFN-alpha resulted in the efficient treatment of SARS-CoV-2 infections in vivo, most probably by the synergistic inhibition of TMPRSS2, a cellular factor essential for viral replication [8].

We uploaded the published information to our web-based drugvirus.info server, which allows tracking the developmental progress of BSAs and BCCs and analyzing the antiviral activities of potent BSAs and BCCs [11,12].

Underlying noncommunicable diseases of infected people and their treatments could modulate virus–host interactions. Petakh et al. proposed that metformin could reduce lung damage and improve the course of the COVID-19 disease in patients with type 2 diabetes [13]. Another study indicated that commonly prescribed drugs modulate influenza A virus–host cell interactions [14].

Thus, our Special Issue highlighted the development of BSAs and BCCs as well as analyzed effects of other drugs and underlying conditions on virus–host interactions. These booming directions of research will ultimately lead to breakthroughs in the treatment of viral diseases in the near future.
